# Risk definition and outcomes with the application of the PEGASUS-TIMI 54 trial inclusion criteria to a “real world” STEMI population: results from the Italian “CARDIO-STEMI SANREMO” registry

**DOI:** 10.1186/s12872-020-01780-y

**Published:** 2021-03-18

**Authors:** Federico Sanchez, Valentina Boasi, Matteo Vercellino, Chiara Tacchi, Pierpaolo Cannarile, Nicoletta Pingelli, Dino Perri, Laura Gomez, Stefano Cattunar, Giovanni Mascelli

**Affiliations:** 1 Department of Cardiology, UTIC Ospedale Civile Di Sanremo, ASL1 imperiese, via Giovanni Borea 56, 18038 Sanremo, IM Italy; 2 ASO Alessandria-Ospedale Civile SS. Antonio, Biagio e Cesare Arrigo, Alessandria, AL Italy

**Keywords:** Bleeding risk, Dual antiplatelet therapy, Ischemic risk, STEMI outcome, STEMI registry, Ticagrelor

## Abstract

**Background:**

The PEGASUS-TIMI 54 trial inclusion criteria effectively identified high-risk patients with recent myocardial infarction (MI) who would benefit from continuing dual antiplatelet therapy (DAPT) with ticagrelor for more than 12 months. It is unknown how many real-world patients meet these criteria during the acute phase of ST-elevation MI (STEMI), or the extent to which these criteria predict a patient's risk and prognosis. Study objectives were: (1) determine the proportion of PEGASUS-TIMI 54-like patients (PG-l) in a real-world cohort of patients hospitalized with STEMI and to assess their ischemic and hemorrhagic risk; (2) examine their ischemic and hemorrhagic in-hospital events (major adverse cardiovascular and cerebrovascular events [MACCE] and clinically relevant bleeding); (3) evaluate their long-term outcomes and the impact on the long-term prognosis of the type of DAPT prescribed at discharge.

**Methods:**

This observational study was conducted in 1086 patients admitted to hospital with a diagnosis of STEMI between February 2011 and March 2018 and enrolled in the CARDIO-STEMI Sanremo registry. Patients’ demographic and clinical characteristics, procedural variables, and individual ischemic and hemorrhagic risk scores were assessed in-hospital. Four-year survival was also analyzed.

**Results:**

The proportion of PG-I patients was 69.2%. Compared with non-PG-l patients, PG-l patients were older, had more multivessel disease and comorbidities, and experienced more frequent MACCE (8.3% vs. 3.6%, *p* = 0.005) and clinically significant bleeding events (6.7% vs. 2.7%, *p* = 0.008), a higher rate of in-hospital death (6.5% vs. 1.5%, *p* < 0.001), and higher follow-up mortality rate (14.8% vs. 7.7%; *p* = 0.002). Four-year survival was significantly lower in the PG-l group (83.9% vs. 91.8%; Log-rank = 0.001) and was related to the cumulative number of concurrent risk factors. In the unadjusted analysis, survival was greater in patients discharged on ticagrelor than on another P2Y_12_ inhibitor (90.2% vs. 76.7%, Log-rank = 0.001), and the difference was particularly evident in PG-l patients.

**Conclusions:**

The risk of MACCE for PG-l patients increased with the number of concurrent PEGASUS-TIMI 54 risk features. Treatment with ticagrelor on discharge was associated with improved survival rates during 4 years of follow-up.

## Background

In Western Europe, 32.8% of patients hospitalized for acute coronary syndrome (ACS) have ST-segment elevation myocardial infarction (STEMI) [1]. According to current guidelines, STEMI patients should receive dual antiplatelet therapy (DAPT) with aspirin (ASA) and a P2Y_12_ inhibitor (with ticagrelor or prasugrel preferred over clopidogrel) for at least 12 months, regardless of whether or not patients underwent percutaneous coronary intervention (PCI) [2, 3]. DAPT prevents the recurrence of ischemic events such as myocardial infarction (MI), stroke, or death from cardiovascular (CV) causes unrelated to the treated coronary lesion, which may occur beyond 1 year after coronary stenting [4, 5]. However, the clinical benefit of continued treatment with P2Y_12_ inhibitors is tempered by a higher risk of bleeding [4, 6], which is correlated with treatment duration [7–14]. The clinical trials ARCTIC-Interruption [15], DAPT [4], DES-LATE [16], OPTIDUAL [17], and meta-analyses [18, 19], showed that the benefit of reduced ischemic events associated with prolonging DAPT beyond 12 months was counterbalanced by an increase in bleeding risk, although study populations were heterogeneous, so no definitive conclusion about the optimal duration of DAPT could be reached. While it has been proposed that this risk could be mitigated in patients with high bleeding risk by a shorter DAPT duration of at least 6 months [3, 20–22], evidence from the PEGASUS-TIMI 54 (Prevention of Cardiovascular Events in Patients with Prior Heart Attack Using Ticagrelor Compared to Placebo on a Background of Aspirin) trial suggested a benefit with prolonged DAPT in patients with high ischemic risk and prior MI [7].

The PEGASUS-TIMI 54 study evaluated the use of DAPT beyond 12 months in 21,162 patients who had had an MI 1 to 3 years prior to enrolment and had a high ischemic risk [23]. The study inclusion criteria also required patients to be ≥ 50 years of age and to have ≥ 1 of the following additional risk factors: age ≥ 65 years, diabetes mellitus, recurrent MI, multivessel coronary artery disease (CAD) or impaired renal function (creatinine clearance < 60 mL/min) [23]. Patients received ASA 75–150 mg/day and add-on ticagrelor 60 or 90 mg twice daily or placebo, and were followed for a median of 33 months [24]. Compared with placebo, ticagrelor was associated with a reduced risk of CV death, MI, or stroke over 3 years and a neutral effect on overall mortality [24]. The two ticagrelor doses were associated with an increased risk of Thrombolysis in Myocardial Infarction (TIMI) major bleeding compared with placebo but the risk of fatal bleeding or intracranial hemorrhage did not differ between ticagrelor and placebo [24]. The benefit of ticagrelor was consistent across the major clinical subgroups of the PEGASUS-TIMI 54 population, 53.4% of whom had experienced a STEMI [24].

Based on the favorable benefit-risk profile, long-term ticagrelor may represent an attractive option, although the benefits seen in the PEGASUS-TIMI 54 trial may not extend to patients at low risk of ischemic events [25, 26] or to patients with a high risk of bleeding, since patients with recent bleeding or requiring oral anticoagulation were excluded from the study [25–27]. The 2017 guidelines of the European Society of Cardiology (ESC) recommend that physicians consider extending DAPT with ticagrelor 60 mg twice daily + ASA beyond 1 year in patients who have tolerated DAPT for 12 months without bleeding, and using clopidogrel or prasugrel as an alternative choice if ticagrelor is not feasible or tolerated [2, 3].

The PEGASUS inclusion criteria may help clinicians to promptly identify patients at high ischemic risk [24], because the number of risk factors present in an individual patient is closely related to the risk of a major adverse cardiac event during follow up [28]. Our hypothesis is that the inclusion criteria in the PEGASUS-TIMI 54 trial could be a useful tool for the identification of high-risk patients, defined as PEGASUS-like (PG-l) patients, immediately after STEMI and for prognosis estimation and therapeutic decision-making. The use of such a tool, to the best of our knowledge, has never been tested.

The aim of the study was (1) to evaluate the proportion of STEMI patients enrolled in the Italian CARDIO-STEMI Sanremo registry who met the PEGASUS-TIMI 54 trial inclusion criteria (PG-l patients) during hospitalization; (2) to evaluate the ischemic risk (using the GRACE score [29]) and the hemorrhagic risk (using the PRECISE-DAPT [21] and CRUSADE [30] scores) of these patients and the incidence of hospital events (death, major adverse CV or cerebrovascular event [MACCE], any clinically significant bleeding based on Bleeding Academic Research Consortium (BARC) type ≥ 2); and (3) to evaluate the overall long-term survival in PG-l and non-PG-l patients and the impact of the type of DAPT on the long-term prognosis in these groups.

## Methods

This study used data from the CARDIO-STEMI Sanremo registry, a single-center, observational study that was conducted at the Sanremo Hospital (Italy) according to national and international guidelines. The protocol was compliant with ethical standards and privacy rule requirements for research and approved by the local ethics committee (Comitato Etico Regione Liguria based at the IRCCS San Martino of Genoa; resolution number 039REG2016). Each patient in the registry provided written informed consent for participation, including consent to be contacted for follow-up information and for their data to be included in subsequent analyses.

All the consenting patients admitted to the hospital with a diagnosis of STEMI between February 2011 and March 2018 were enrolled; for every patient, demographic and clinical characteristics were recorded and individual ischemic risk factors (i.e. age, diabetes mellitus, a prior spontaneous MI, multivessel CAD, creatinine clearance < 60 mL/min) and hemorrhagic risk scores were assessed during hospitalization; TIMI flow grade (before and after PCI), the rate of in-hospital MACCE, ST segment resolution, bleeding, and survival were also collected. MACCE were defined according the PLATO trial criteria [11]; MI was defined according to the fourth universal definition of MI [31]; bleeding was assessed following the standardized definition provided by Mehran et al*.* [32]. Hemorrhagic risk factors were assessed using the CRUSADE [30] and PRECISE-DAPT [21] scores; the GRACE risk score was used to assess ischemic risk [29]. The PRECISE DAPT score predicts the risk of bleeding in patients treated with DAPT; a PRECISE DAPT score above the recommended cut-off point (≥ 25) identifies patients who might benefit from DAPT for < 12 months [21]. Patients were treated according to the prevailing guidelines at the time of their enrolment, all of which advised continuation of DAPT for at least 1 year after discharge in patients who had undergone PCI. Follow-up was performed through a telephone interview with the patient or the patient’s family physician, with the support of the local health registry.

Patients were categorized into two cohorts, depending on whether their demographic and clinical characteristics were consistent with the inclusion criteria for the PEGASUS-TIMI 54 study (age ≥ 50 years and ≥ 1 high-risk criterion). Patients who met these criteria were designated PEGASUS-like (PG-l) and all other patients were designated as non-PG-l patients. Comparisons were made between the characteristics treatment patterns, and outcomes in the two groups.

### Statistical analyses

The patients’ baseline demographic and clinical characteristics were reported using relevant descriptive statistics (means ± standard deviation [SD], median and interquartile range [IQR], or percentage). Kolmogorov–Smirnov tests were used to assess the normal distribution of continuous variables; those with skewed distribution were compared using the Mann–Whitney U test. Categorical variables and continuous variables that were not normally distributed were compared using the χ^2^ test and Kruskal–Wallis test, respectively. Medians across groups were compared using a Median test for two independent medians.

A stepwise block Cox multivariate analysis was used to identify predictors of long-term mortality, with age, diabetes, PCI, ≥ 50% ST resolution, renal function, ejection fraction and therapy at discharge included as candidate variables.

For all tests, a *p* value < 0.05 was considered significant. Survival analyses were generated using the Kaplan–Meier method and compared with the log-rank test. Statistical analyses were undertaken using SPSS version 22.0 (SPSS Inc., Chicago, IL, USA).

## Results

The demographic and baseline data for the 1086 patients (73.5% males) enrolled in the CARDIO-STEMI Sanremo registry are shown in Table 1. Median age (range) of patients was 66 (56–77) years, with 90.3% of the patients aged ≥ 50 years and 55.3% aged ≥ 65 years. Overall, 17.4% of patients had diabetes, 9.8% had previous MI, 40.5% had multivessel CAD, and 28.3% had impaired renal function. Only 5% of patients had experienced a previous hemorrhage.

**Table 1:** Baseline and demographic data for STEMI patients

	STEMI patients (*n* = 1086)
Median (IQR) age, years	67 (56–77)
Age categories	
≥ 50 years	981 (90.3)
≥ 65 years	601 (55.3)
≥ 75 years	335 (30.8)
≥ 85 years	81 (7.5)
Sex (male)	798 (73.5)
Median (IQR) BMI, kg/m^2^	26 (24–29)
Diabetes mellitus	189 (17.4)
GFR < 60 mL/min/1.73m^2^	301 (28.3)
Multivessel CAD	440 (40.5)
Previous AMI	106 (9.8)
Previous bleeding	54 (5.0)
Previous neoplasia	146 (13.5)
TIA	27 (2.5)
Stroke	37 (3.4)
Hypertension	605 (55.7)
Dyslipidemia	379 (34.9)
Smokers	436 (40.1)
Ex-smokers	234 (21.5)
Family history of CAD	237 (21.8)

Data are expressed as n (%) unless otherwise stated

*AMI* acute myocardial infarction, *BMI* body mass index, *CAD* coronary artery disease, *GFR* glomerular filtration rate, *IQR* interquartile range, *STEMI* ST-elevation myocardial infarction, *TIA* transient ischemic attack

The PEGASUS-TIMI 54 inclusion criteria (age ≥ 50 years and ≥ 1 high-risk criterion) were met by 751 (69.2%) patients (PG-l patients). The demographic and baseline data in the PG-l population and the non-PG-l population are shown in Table 2. The PG-l population was older than the non-PG-l group, with 74.2% of PG-l patients being ≥ 65 years old and 41.1% being ≥ 75 years old (*p* < 0.001). Hypertension was more prevalent among PG-l patients (61.9% vs. 41.8%, *p* < 0.001), while no significant difference was found between the two groups for dyslipidemia. Non-PG-l patients were twice as likely to be smokers and to have a family history of CAD compared with PG-l patents, who included a higher proportion of patients with impaired renal function (38.2% vs. 6.6%; *p* < 0.001) and multivessel CAD (52.3% vs. 14.0%; *p* < 0.001).

**Table 2:** Baseline and demographic data for non-PG-l vs. PG-l patients

	Non-PG-l patients (n = 335)	PG-l patients (n = 751)	*P* value
Median (IQR) age, years	54 (49–59)	71 (63–79)	< 0.001
Age ≥ 65 years	44 (13.1)	557 (74.2)	< 0.001
Age ≥ 75 years	26 (7.8)	309 (41.1)	< 0.001
Sex (male)	227 (82.7)	521 (69.4)	< 0.001
Median (IQR) BMI, kg/m^2^	27 (24–29)	26 (24–29)	0.008
Diabetes mellitus	21 (6.3)	168 (22.4)	< 0.001
GFR < 60 mL/min/1.73m^2^	22 (6.6)	279 (38.2)	< 0.001
Multivessel CAD	47 (14.0)	393 (52.3)	< 0.001
Previous bleeding	54 (16.2)	0	< 0.001
Previous AMI	16 (4.8)	90 (12.0)	< 0.001
Previous neoplasia	29 (8.7)	117 (15.6)	0.002
TIA	6 (1.8)	21 (2.8)	0.326
Hypertension	140 (41.8)	465 (61.9)	< 0.001
Dyslipidemia	109 (32.5)	270 (36.0)	0.276
Smokers	202 (60.3)	234 (31.2)	< 0.001
Ex-smokers	42 (12.5)	192 (25.6)	< 0.001
Family history of CAD	112 (33.4)	125 (16.6)	< 0.001

Data are expressed as n (%) unless otherwise stated

*AMI* acute myocardial infarction, *BMI* body mass index, *CAD* coronary artery disease, *GFR* glomerular filtration rate, *IQR* interquartile range, *PG-l* PEGASUS-TIMI 54-like patients, *TIA* transient ischemic attack

The GRACE and CRUSADE scores were significantly higher in the PG-l than the non-PG-l cohort (114 [95–132] vs. 77 [63–90], *p* < 0.001, and 28.5 [19–39] vs. 16 [9–23], *p* < 0.001, respectively). In our study, the fraction of patients at high bleeding risk (PRECISE DAPT score ≥ 25) was also significantly higher in the PG-l group (41.7% vs. 33.2%, *p* = 0.018).

No significant between-group differences were seen for the proportion of patients treated with PCI (90.4% vs. 91.9%, *p* = 0.44) or with ≥ 50% ST resolution, achieved by 81.3% of the PG-l population versus 81.9% of non-PG-l patients (*p* = 0.86). The frequency of thromboaspiration and the use of radial access were similar between groups (37% vs. 33%, *p* = 0.40 and 65.8% vs. 60.1, *p* = 0.12, respectively). Similarly, no significant differences were reported between the PG-l and non-PG-l groups in the proportion of patients presenting with spontaneous reperfusion (initial TIMI flow of ≥ 2 in 41.8% vs. 38.0%, *p* = 0.29) and achieving reperfusion after angioplasty (a final TIMI flow of ≥ 2 in 94.3% vs. 95.0%, *p* = 0.76). The left ventricular ejection fraction during hospitalization was lower in PG-l than non-PG-l patients (median [range] 45% [40–55] vs. 50% [45–55], *p* < 0.001).

In keeping with the high-risk profile of the PG-l cohort (older age, high prevalence of multivessel CAD, higher GRACE and CRUSADE scores, and lower ejection fraction), adverse outcomes during hospitalization were significantly more common in the PG-l group than the non-PG-l group, including BARC ≥ 2 bleeding (6.7% vs. 2.7%, *p* = 0.008) and MACCE, defined as in-hospital all-cause death, AMI, stent thrombosis or acute ischemic stroke and cerebrovascular events (8.3% vs. 3.6%, *p* = 0.005, respectively). Similarly, PG-l patients had a longer hospital stay (median [range] 5 [4–7] days vs. 4 [4, 5] days, *p* < 0.001), and a nearly fivefold higher rate of in-hospital death (6.5% vs. 1.5%, *p* < 0.001).

**Fig. 1:**
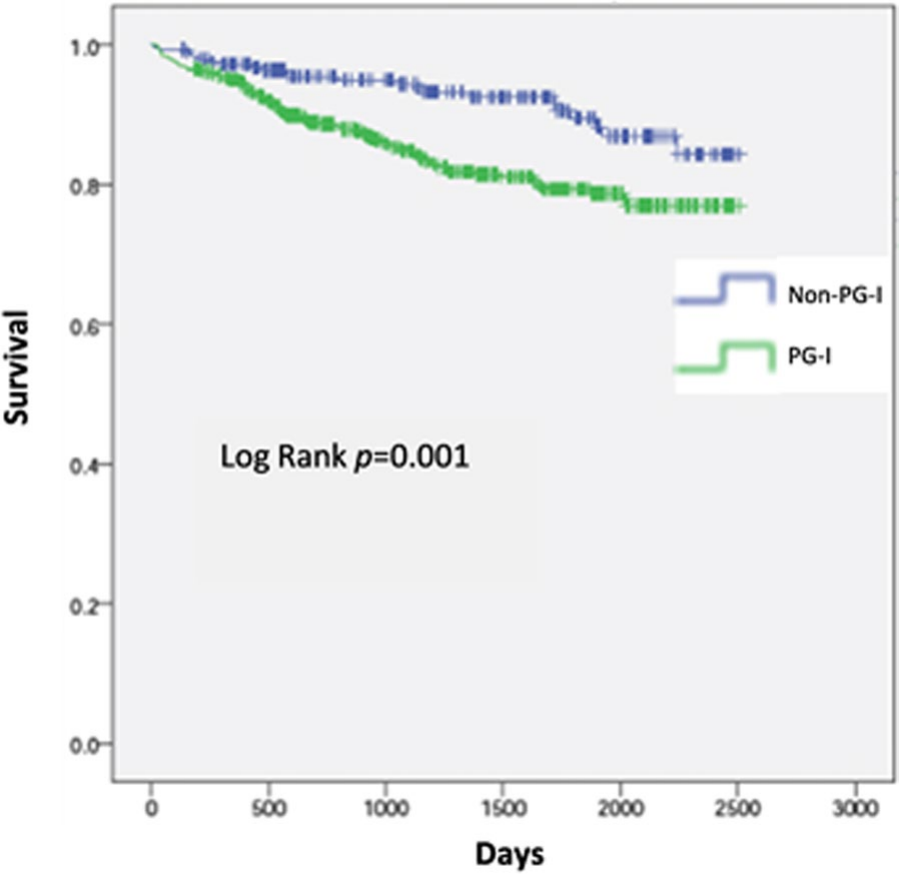
Kaplan–Meier analysis of 4-year survival for PG-l versus non-PG-l patients

**Fig. 2:**
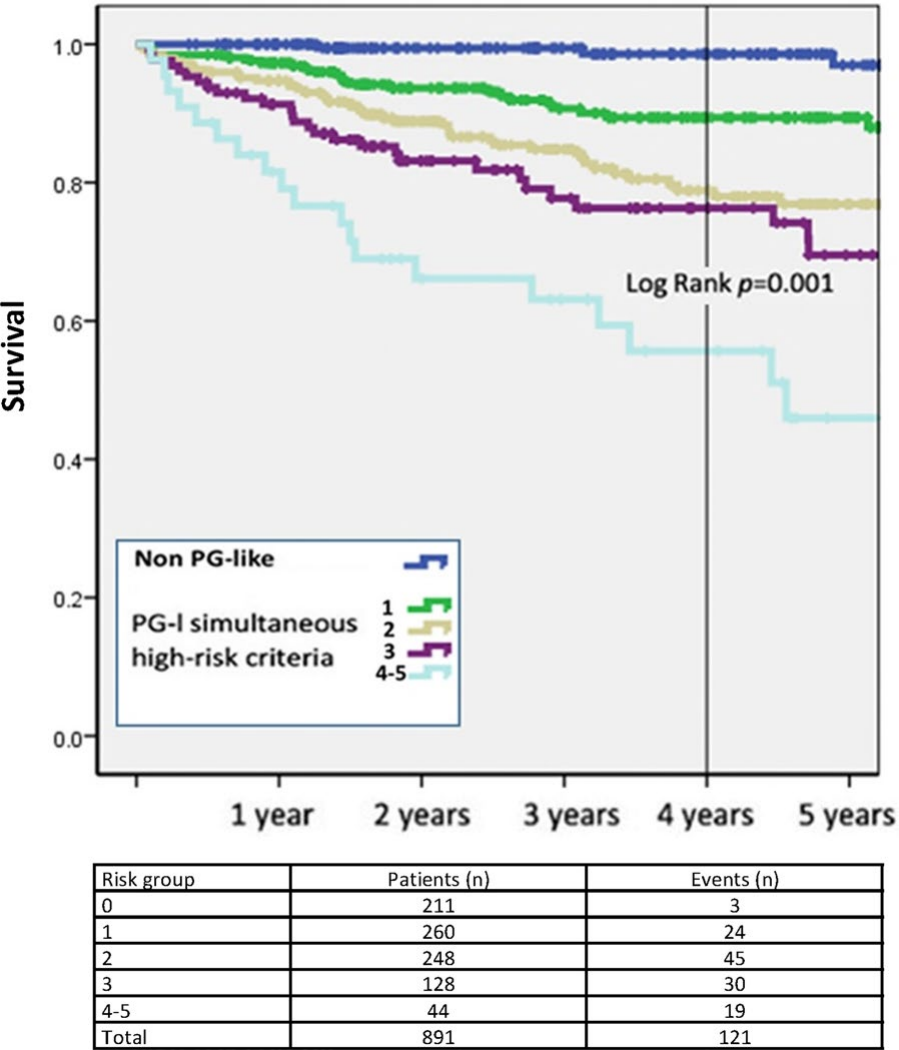
Kaplan–Meier analysis of 4-year survival in the non-PG-l patients (blue line) and the PG-l patients, stratified by the cumulative number of risk factors present. The table shows the number of patients in each risk group. Abbreviation: PG-l = PEGASUS-TIMI 54-like patients

The median (range) long-term follow-up for STEMI patients was 1142 (577–1810) days, and mean ± SD follow-up was 1207 ± 697 days. The Kaplan–Meier estimated 4-year survival rate was significantly lower among PG-l than non-PG-l patients (83.9% vs. 91.8%; Log-rank = 0.001; Fig. 1). The 4-year survival was also dependent on the number of PEGASUS-TIMI 54 high risk criteria that were present (Log-rank = 0.001), with survival decreasing as the number of concomitant risk criteria increased (Fig. 2).

Unless contraindicated, all patients were treated with a P2Y_12_ inhibitor (prasugrel, ticagrelor, or clopidogrel) on a background of ASA (DAPT). The majority of patients were discharged on ticagrelor (52.7%), followed by clopidogrel (28.9%) and prasugrel (12.3%). The discharge therapy with clopidogrel and prasugrel varied significantly with age (*p* = 0.001), with clopidogrel prescribed more frequently for older than younger patients, and prasugrel prescribed more often for younger patients. However, the proportion of patients receiving ticagrelor varied little between age groups. No statistically significant differences were noted in sex, age, frequency of in-hospital bleeding (BARC ≥ 2) or the proportion of patients with ≥ 50% ST resolution between those treated with ticagrelor and those who received clopidogrel or prasugrel. However, patients who received ticagrelor versus another P2Y_12_ inhibitor were more often treated with PCI (97.6% vs. 85.9%; *p* < 0.001) and had a lower follow-up mortality (unadjusted rate 8.10% vs. 19.0%, *p* < 0.001).

**Fig. 3:**
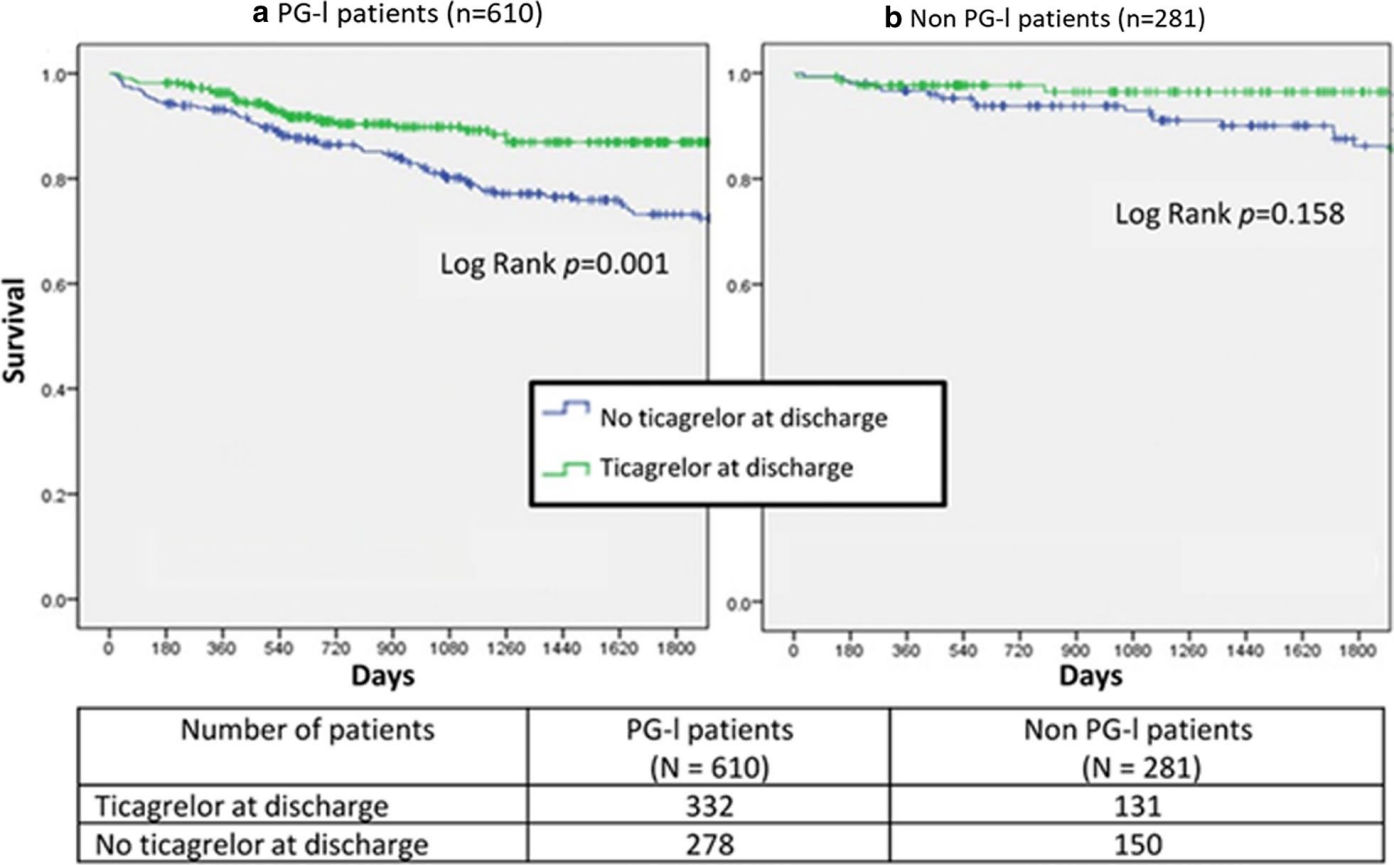
Kaplan–Meier analysis of follow-up survival for (**a**) PG-l and (**b**) non-PG-l patients, stratified by ticagrelor prescription at discharge. The table shows the number of patients on ticagrelor versus no ticagrelor at discharge. Abbreviation: PG-l = PEGASUS-TIMI 54-like patients

In the PG-l group, ticagrelor at discharge was associated with significantly lower mortality during follow-up compared with another P2Y_12_ inhibitor at discharge, with a 4-year survival probability of 90.2% versus 76.7% (Log-rank *p* = 0.001), as shown in the Kaplan–Meier analysis shown in Fig. 3a. There was no statistically significant difference in mortality between ticagrelor and no ticagrelor at discharge among patients in the non-PG-l group (Fig. 3b; *p* = 0.158).

In the stepwise Cox multivariate analysis, ticagrelor therapy at discharge was found to be the only independent predictor of follow-up mortality (Relative Risk [RR] 0.62, 95% Confidence Interval [CI]: 0.39–0.98, *p* = 0.04), and only in the PG-l group, but not in non-PG-l patients (RR 0.70, 95% CI: 0.20–2.40, *p* = 0.57). Although ticagrelor was associated with reduced mortality in the PG-l group vs. the non-PG-l group, the difference was not statistically significant (p for heterogeneity is 0.86); the lack of a significant effect on mortality in the non-PG-l group may be due to the small number of events/wide confidence intervals.

## Discussion

Nearly 70% of the patients admitted to hospital for STEMI met the PEGASUS-TIMI 54 trial inclusion criteria. When compared with non-PG-l patients, PG-l patients were at increased risk of in-hospital and long-term mortality; they were older, with lower ejection fraction, more complex CAD, and in some cases had one or more comorbid conditions. The GRACE score was significantly higher in the PG-l cohort, who experienced a longer hospital stay and an almost five times higher rate of in-hospital death. The 4-year survival of the PG-l population was significantly lower and positively related to the number of associated PEGASUS-TIMI 54 high risk factors: the higher the number of concurrent risk factors, the higher the mortality. This is consistent with previous research by Parodi et al. in similar analyses of patients with ACS and PCI followed for up to 3 years [28]. We can hypothesize that a simple association of risk factors, or their inclusion within more complex scores, could adequately identify patients with a more favorable net clinical benefit from prolonged DAPT. This could translate into a new simple bedside risk-assessment tool based on the PEGASUS-TIMI 54 criteria for the risk stratification of patients with acute MI at index hospitalization.

By adopting the PEGASUS-TIMI 54 criteria we have also been able to identify a population of patients with a high risk of bleeding: the PG-l cohort had a significantly higher CRUSADE score, significantly more patients with a PRECISE-DAPT score > 25, and a significantly greater incidence of in-hospital BARC ≥ 2 bleeding compared with the non-PG-l cohort. While the risk of bleeding was increased in the PG-I cohort, it is worth bearing in mind that interpreting this risk must be balanced against the level of ischemic risk. As shown in the PRECLUDE study [33], prior bleeding is the best predictor of bleeding, so if prior major bleeding is excluded, patients with ischemic risk factors are more likely to have a new ischemic event than bleeding. This suggests that, while our PG-I cohort might have had an increased risk of bleeding, their risk of ischemic occurrence may be greater. The trade-off between ischemic benefit and bleeding risk is of paramount importance for cardiologists who need to assess therapies and manage patients, and the higher bleeding risk associated with DAPT is often outweighed by the benefits of a reduced risk of ischemic events. Considering that bleeding and ischemic risks are often strongly correlated, risk scores enable the stratification of patients and may facilitate tailored decision-making on the type and duration of DAPT [34, 35]. Current ESC recommendations for long-term secondary prevention with DAPT acknowledge this delicate balance of risk, and recommend DAPT for patients at high or moderately increased ischemic risk without high bleeding risk [36].

Among patients discharged on DAPT, prasugrel was preferentially used in younger patients whereas treatment with clopidogrel was more frequent among elderly patients. In contrast, the proportion of patients discharged on ticagrelor did not differ with respect to age, showing that, in the real-life clinical practice of this hospital setting, physicians consider ticagrelor a safe therapeutic option. Indeed, the PLATO study had already shown better follow-up survival for patients treated with ticagrelor versus clopidogrel [11]; a similar benefit in a real-world STEMI population had also been highlighted by our study group [37].

Finally, although results show that ticagrelor was associated with lower mortality rates for the next 12 months compared with other antiplatelet agents, the difference was statistically significant only for PG-l patients (unadjusted analysis). A possible explanation for this is the small number of patients in the non-PG-l group, limiting the power of that analysis. For PG-l patients, the benefit of therapy with ticagrelor 90 mg twice daily persisted for the first 12 months, was sustained over time and was appreciable also at long term follow-up.

From a practical point of view, the better survival among patients receiving ticagrelor could be explained as an extended clinical benefit of ticagrelor in the long term, compared with other antiplatelet agents, which might be due to the decrease in the risk of thrombotic events, without a concomitant increase in the risk of major bleeding, consistent with the findings from the PLATO study and from the sub-study in the STEMI population [11, 38].

### Study limitations

This is a real-life study performed on data independently gathered from more than 1000 patients enrolled in a single-center, observational study, with an average 4-year follow-up, and is affected by the inherent limitations of a non-randomized prospective study. No MACCE or bleeding events were recorded after the hospital phase. This limitation may have affected the recording of any favorable effect on the rate of MACCE occurring only after 30 days [24]. However, this is a frequent limitation of hospital registry data. Our study did collect long-term mortality data, which can be considered as a surrogate measure of post-discharge complications including MACCE and bleeding. Another limitation of this study is the lack of data collection on hemorrhagic events during the first 12 months of follow-up; therefore, we could not establish the proportion of patients eligible for prolonged use of DAPT beyond 12 months. Nor did we collect data on use of DAPT beyond 12 months. A history of bleeding was an exclusion criterion for the PEGASUS-TIMI 54 trial. This study lacks the statistical power to show a reduction in mortality for non-PG-l patients over the 4-year follow-up. Finally, the potential for confounding by measured or unmeasured variables cannot be ruled out.

## Conclusions

Nearly 70% of the patients enrolled in the CARDIO-STEMI Sanremo registry match the PEGASUS-TIMI 54 inclusion criteria, which successfully identified a population of real-world STEMI patients at a high risk of MACCE or death. When compared with non-PG-l patients, the prognosis for PG-l patients, who are older, and may have one or more comorbid conditions, is worse during both short- and long-term follow-up, and the risk of an adverse outcome progressively increases with the number of concurrent PEGASUS-TIMI 54 high-risk features that are present. Thanks to their ease of use in daily clinical practice, the PEGASUS-TIMI 54 inclusion criteria, along with other scores such as the DAPT score and the PRECISE-DAPT score, should become a more frequently used tool to support clinical decision-making about the duration of DAPT. Notwithstanding the limitations of this real-life registry study, our data also suggest that treatment with ticagrelor on discharge is associated with improved 4-year survival rates in patients who meet the criteria for PEGASUS-TIMI 54 inclusion.

## Data Availability

The datasets used and/or analysed during the current study are available from the corresponding author on reasonable request.

## Abbreviations

ACS: Acute coronary syndrome
ASA: Aspirin
BARC: Bleeding Academic Research Consortium
CV: Cardiovascular
CI: Confidence interval
CAD: Coronary artery disease
DAPT: Dual antiplatelet therapy
ESC: European Society of Cardiology
IQR: Interquartile range
MACCE: Death, major adverse CV or cerebrovascular event
MI: Myocardial infarction
PG-l: PEGASUS-like
PEGASUS-TIMI 54: Prevention of Cardiovascular Events in Patients with Prior Heart Attack Using Ticagrelor Compared to Placebo on a Background of Aspirin
RR: Relative risk
SD: Standard deviation
STEMI: Segment elevation myocardial infarction
TIMI: Thrombolysis in Myocardial Infarction
